# Persistence of lung structural and functional alterations at one year post‐COVID‐19 is associated with increased serum PD‐L2 levels and altered CD4/CD8 ratio

**DOI:** 10.1002/iid3.1305

**Published:** 2024-07-19

**Authors:** Ivette Buendia‐Roldan, Karen Martínez‐Espinosa, Maria‐Jose Aguirre, Hiram Aguilar‐Duran, Alexia Palma‐Lopez, Yadira Palacios, Andy Ruiz, Lucero A. Ramón‐Luing, Ranferi Ocaña‐Guzmán, Gloria Pérez‐Rubio, Ramcés Falfán‐Valencia, Moisés Selman, Leslie Chavez‐Galan

**Affiliations:** ^1^ Instituto Nacional de Enfermedades Respiratorias Ismael Cosío Villegas Mexico City Mexico

**Keywords:** biomarkers, COVID‐19, follow‐up, interstitial lung, post‐COVID‐19, soluble PD‐L2

## Abstract

**Background:**

Persistent respiratory symptoms and lung abnormalities post‐COVID‐19 are public health problems. This study evaluated biomarkers to stratify high‐risk patients to the development or persistence of post‐COVID‐19 interstitial lung disease.

**Methods:**

One hundred eighteen patients discharged with residual lung abnormalities compatible with interstitial lung disease (COVID‐ILD patients) after a severe COVID‐19 were followed for 1 year (post‐COVID‐ILD patients). Physical examination, pulmonary function tests, and chest high‐resolution computed tomography (HRCT) were performed. Soluble forms (s) of PD‐L1, PD‐L2, TIM‐3, and GAL‐9 were evaluated in serum and cell culture supernatant, as well as T‐cells subsets and the transmembrane expression of PD‐L1 and PD‐L2 on the cell surface.

**Results:**

Eighty percent of the post‐COVID‐ILD patients normalized their lung function at 1‐year follow‐up, 8% presented COVID‐independent ILD, and 12% still showed functional and HRCT alterations. PD‐L2 levels were heterogeneous during acute COVID‐19 (aCOVID); patients who increased (at least 30%) their sPD‐L2 levels at 1 year post‐COVID‐19 and exhibited altered CD4/CD8 ratio showed persistence of chest tomographic and functional alterations. By contrast, patients who decreased sPD‐L2 displayed a complete lung recovery. sPD‐L1, sTIM‐3, and sGAL‐9 increased significantly during aCOVID and decreased in all patients after 1‐year follow‐up.

**Conclusion:**

Increased sPD‐L2 and an altered CD4/CD8 ratio after 12 months of aCOVID are associated with the persistence of lung lesions, suggesting that they may contribute to lung damage post‐COVID‐19.

## Graphical abstract

Increased sPD‐L2 and an altered CD4/CD8 ratio after 12 months of acute COVID‐19 (aCOVID) are associated with the persistence of lung lesions. sPD‐L1 could be useful to improve the diagnosis during aCOVID.

## 1 INTRODUCTION

The spread of SARS‐CoV‐2, the etiological agent of the current pandemic Coronavirus Disease 2019 (COVID‐19), remains a public health problem. WHO reports indicate that on January 19, 2024, there were 774,075,198 confirmed COVID‐19 cases worldwide, and 7,012,984 people died from COVID‐19.^1^

Diverse sequelae by severe COVID‐19 have been reported, but there is no global consensus on the clinical terminology during the follow‐up of COVID‐19 survivors; expressions such as “post‐COVID‐19 syndrome,” “long‐term COVID‐19,” or “long COVID” are used; the last is defined as a debilitating illness that occurs in at least 10% of patients after a severe SARS‐CoV‐2 infection and more than 200 symptoms have been identified.^2^ Although most patients improve symptoms and exercise capacity over time, a subgroup shows persistent lung physiological and radiographic changes at 12 months post‐COVID‐19.^3^

The prevalence of the persistence of respiratory symptoms and/or radiological lung abnormalities is yet unclear; some authors called these abnormalities post‐COVID‐19 lung fibrosis. However, this could be an overstatement because many of the changes are temporary and tend to be resolved on follow‐up; thus, other authors propose using the term “post‐COVID‐19 interstitial lung changes” (post‐COVID‐ILD).^4^ Different associations have been reported with hematologic, biochemical, coagulation, inflammatory, and potential new biomarkers in disease progression,^5^ but information related to long‐term sequelae is still limited. Consequently, stratifying high‐risk patients to develop true interstitial lung damage post‐COVID‐19 is essential. Identifying blood biomarkers could help establish an early treatment scheme.

In this regard, serum levels of the molecules programmed cell death ligand 1 (PD‐L1), mucin domain‐containing molecule‐3 (TIM‐3), and its ligand Galectin‐9 (GAL‐9) have been evaluated in the context of diverse viral infections, mainly in those where there is an excessive inflammatory response, including SARS‐CoV‐2 infection.^6^, ^7^, ^8^, ^9^ The expression of PD‐L1 in COVID‐19 patients has been evaluated at protein and transcriptional levels, including the expression of its receptor PD‐1 on the circulating cells.^10^, ^11^, ^12^ A report proposed that T‐cells from symptomatic post‐COVID‐19 patients are PD‐1+ and exhibit exhausted function, suggesting an abnormal function of the PD‐1/PD‐L1 immune checkpoint axis.^13^

Even though the PD‐L1‐mediate axis plays a critical role in the immune response against SARS‐CoV‐2, PD‐L2 has not been intensely studied in COVID‐19 and post‐COVID‐19 contexts. PD‐L2 is also a molecule involved in immune regulation, and its function is mediated by interaction with the same receptor, PD‐1.^14^, ^15^

This study aimed to evaluate the clinical outcome (at 1 year post‐COVID‐19) of patients discharged from the hospital after a severe COVID‐19 and exhibiting interstitial lung disease (ILD) to identify a potential biomarker of the persistence of lung damage. Importantly, all patients in this study required invasive mechanical ventilation (IMV) at acute COVID‐19 time (aCOVID). They were discharged with respiratory symptoms and/or radiological lung alterations evocative of the development of residual lung abnormality by COVID‐19, and abnormalities were compatible with ILD, including ground‐glass opacities (GGOs), subpleural reticulations, cystic changes, traction bronchiectasis, parenchymal bands and/or architectural distortion). After 1 year of the aCOVID, patients with post‐COVID‐ILD were clinically evaluated, and serum levels of PD‐L1, PD‐L2, TIM‐3, and GAL‐9, as well as transmembrane expression of the PD/PD‐L axis on the T‐cells surface, and molecules involved in the function of T‐cells, were evaluated.

## 2 METHODS

### 2.1 Ethical approval

This protocol was approved by the ethical committee of the Instituto Nacional de Enfermedades Respiratorias Ismael Cosío Villegas (INER, Protocol numbers C53‐20 and B04‐22). All individuals signed a consent letter to participate in this study. All procedures were performed in agreement with the 1964 Helsinki Declaration and the ethical standards of the Institutional Ethics Committees.

### 2.2 Study design and participants

**Figure 1:**
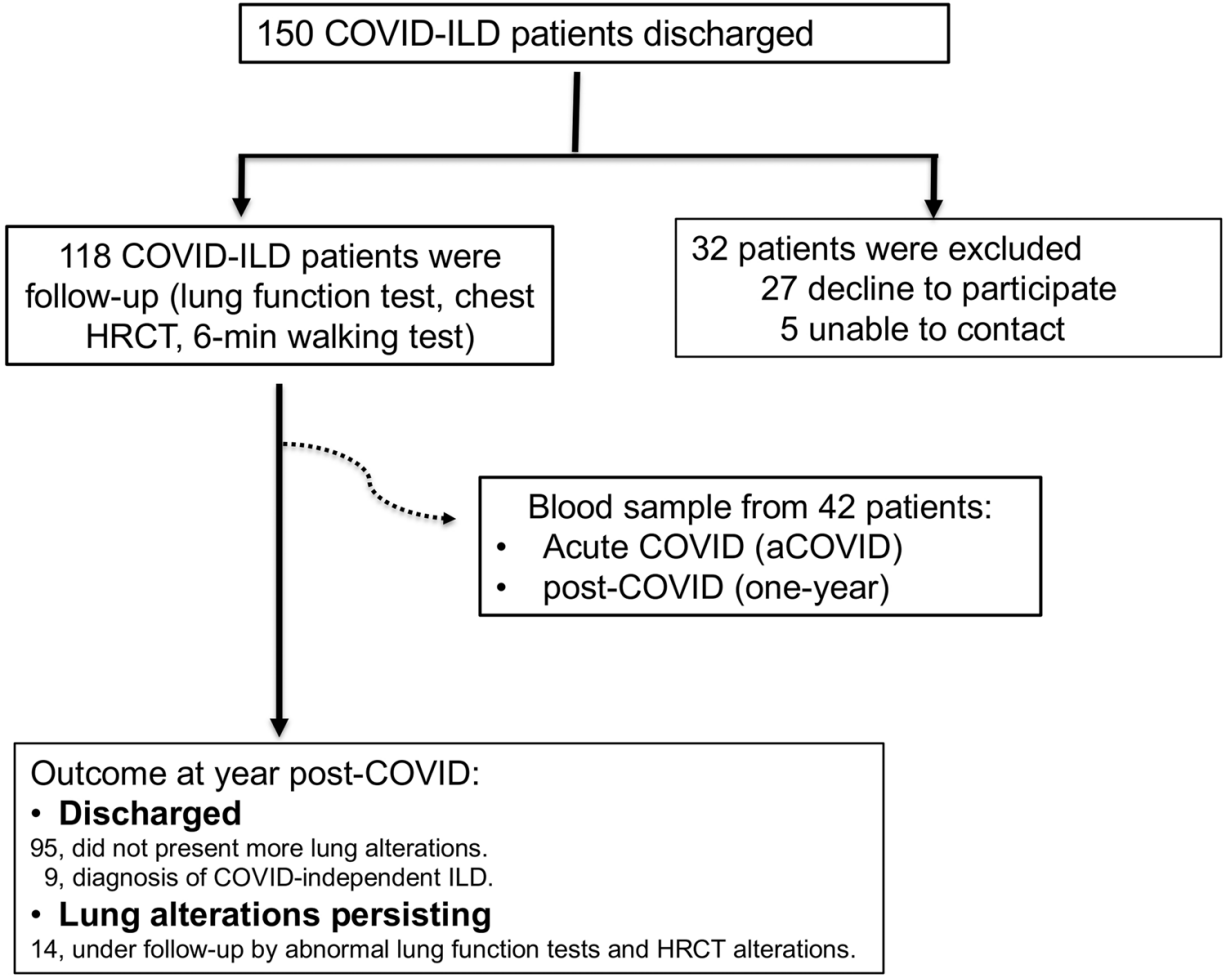
Workflow of enrolled patients. One hundred fifty subjects had severe acute COVID‐19 (aCOVID), and they were discharged from the hospital exhibiting COVID‐associated interstitial lung disease (ILD); from them, 118 were accepted to be clinically evaluated after 1 year of aCOVID (post‐COVID‐ILD). From the total patients, serum from 42 patients (indicated as a dotted line) matched at both their aCOVID and post‐COVID‐ILD time were used to evaluate levels of soluble molecules, and cells were used to evaluate a phenotypical characterization. In 1 year, the clinical evaluation of 118 post‐COVID‐ILD patients indicated that they had diverse outcomes, according to the ILD evolution. HRCT, high‐resolution computed tomography.

One hundred fifty patients were discharged (after a severe COVID‐19) with lung abnormalities compatible with ILD at INER in April–July 2020 and contacted by phone to be invited to participate. Twenty‐seven patients refused to participate, and five could not attend the new appointment. Thus, this study included 118 COVID‐19 patients who attended the follow‐up visit 1 year after discharge (post‐COVID‐ILD), where pulmonary function tests, high‐resolution computed tomography (HRCT), and a 6‐min walking test were performed (Figure 1).

From the 118 post‐COVID‐ILD patients, we obtained blood samples from 42 when they had aCOVID, obtained during the first day of the hospitalization (this sample was preserved in our biobank), and the blood sample at post‐COVID‐19 time. The complete cohort (118 patients) was clinically evaluated to obtain the final diagnosis of interstitial lung damage after 1 year of the aCOVID. Thus, 95 did not display lung functional alterations; nine patients received an ILD diagnosis but COVID‐19‐independent (four with autoimmune lung disease, two with idiopathic pulmonary fibrosis, one with combined pulmonary fibrosis and emphysema, and one with pulmonary alveolar proteinosis. Another patient had chronic obstructive pulmonary disease). All of them were transferred to the corresponding Clinics to receive the appropriate treatment. Currently, 14 post‐COVID‐ILD patients continue under the follow‐up of this protocol because they continue showing abnormal lung function tests and HRCT alterations (Figure 1).

During the hospitalization, patients had severe COVID‐19. For the diagnosis, the clinician staff considered clinical signs of pneumonia (fever, cough, dyspnea, and tachypnea) plus any of the following: respiratory rate >30 inspirations/min, severe respiratory distress, SpO_2_ < 90% on room air, arterial oxygen pressure/fraction of inspiration of O_2_ (PaO_2_/FiO_2_) < 100 mmHg, required IMV, and RT‐PCR confirmed diagnosis for SARS‐CoV2. Clinical data for the acute phase were retrieved from electronic medical records, including demographic characteristics (age, sex, education, and smoking), clinical characteristics (comorbidities, time of onset of symptoms, and chest images), laboratory test results, and treatment (corticosteroids, antibiotics, and antivirals including lopinavir‐ritonavir and hydroxychloroquine).

Blood samples from two extra groups were obtained as controls. One group included 32 healthy donors (hereafter called HD). For the second group, a blood sample was obtained from 22 post‐COVID‐19 patients; we did not have their blood sample during their aCOVID, but according to the electronic medical records, they had a similar clinical condition to the patient group post‐COVID‐ILD, and they were discharged without ILD. This group was considered a verification cohort of the post‐COVID‐19 behavior (hereafter called VC). Both control groups were matched by age with the COVID‐ILD group.

### 2.3 Lung function and CT score

Pulmonary function tests were performed according to the statement of the American Thoracic Society with an Easy One Pro‐Lab®; the following parameters were measured: forced vital capacity (FVC), volume expired in the first second (FEV1), and the relationship FEV1/FVC. Diffusing capacity of the lungs for carbon monoxide (DL_CO_) was measured using the unique respiration test, and the altitude corrected the value of the predicted percentage. For the 6 MW test, we used a NONIN® pulse oximeter. The results were expressed as meters and percentage of oxygen saturation at the test's beginning and end.

All patients underwent HRCT at COVID‐19 and 1 year after COVID‐19. The procedure was performed in the imagenology department at INER and evaluated by an experienced radiologist and pulmonologist. To obtain a parameter of the extent of the compromised lung, we used a semi‐quantitative score previously reported^16^; hereafter, it is called a CT score.

This CT score was calculated for each of the five lobes, considering the extent of anatomical involvement, as follows: 0, no involvement; 1, less than 5% participation; 2, a commitment of 5%–25%; 3, involvement of 26%–50%; 4, participation from 51% to 75%; and 5, more than 75% participation. The total CT score is the summary of each lobe score.

### 2.4 Blood sample and mononuclear cells

The blood sample was collected using BD Vacutainer tubes (BD Biosciences). The clinical laboratory staff collected one blood sample at the hospital admission time for initial clinical analysis (considered the aCOVID sample) and another at 1‐year post‐COVID‐19. Mononuclear cells were isolated by standard Lymphoprep™ (Accurate Chemical‐Scientific) centrifugation gradient within 1 h of the blood draw and were subsequently cryopreserved. Plasma was obtained and stored at −20°C until use.

### 2.5 Flow cytometry

Cells were incubated with fluorochrome‐conjugated monoclonal antibodies (mAbs) against CD3, CD4, CD8, CD45RA, CCR7, CD28, PD‐1, PD‐L1, PD‐L2, Granzyme B, Perforin, TNF, and IFN‐γ (BioLegend); more details about mAbs are described in Supporting Information S1: Table S1.

First, cells were incubated for 20 min at 4°C in a staining buffer (BioLegend) with mAbs CD3, CD4, CD8, CD45RA, CCR7, CD28, PD‐1, PD‐L1, PD‐L2 for the extracellular phenotype. Posteriorly, cells were fixed and permeabilized with the kit Citofix/Citoperm (Beckton‐Dickinson) following the manufacturer's indications. Then, the mAbs Granzyme, Perforin, TNF, and IFN‐γ were used to identify intracellular molecules. Finally, cells were acquired in a FACS Aria II flow cytometer (Becton Dickinson), single fluorochrome compensation was applied, and Fluorescence‐minus‐one was used as controls. Typically, 100,000 events were acquired.

The analysis was performed using FlowJo software V10.0.8 (Tree Star). The strategy is shown in Supporting Information S1: Figure S1. Briefly, we identified the quality of the events in the time/CD45RA gate; the singlet cells were delimited by their size (FSC: forward scatter) and complexity (SSC: side scatter). We identified the region of mononuclear cells with the FSC and the SSC gate; the expression of CD3+ allowed us to identify T cells; then, in this gate, we identified CD4+ (T helper) and CD8+ (cytotoxic T) lymphocytes; finally, the expression of molecules was identified with the mAbs previously described.

### 2.6 Cell culture

Cells were seeded in 24‐well culture plates (Sarstedt) at a density of 1 × 10^6^ cells/well with RPMI 1640 medium (Sigma‐Aldrich) supplemented with l‐glutamine (2 mM; GIBCO), streptomycin‐penicillin mix solution (Sigma‐Aldrich), and 10% heat‐inactivated fetal bovine serum (GIBCO).

For the cell culture, three different conditions were used: RPMI‐supplemented medium (negative control), anti‐CD3/anti‐CD28 (1 μg/mL) (polyclonally stimulated), and isotype control (IgG2, 1 μg/mL) as the negative control. Six hours before the culture ended, Brefeldin A (1×) was added (Beckton‐Dickinson). Twenty‐four hours post‐culture, cells were recovered for flow cytometry analysis, and the supernatant was frozen at −20°C until use. More details about mAbs used for the culture are described in Supporting Information S1: Table S1.

### 2.7 Enzyme‐linked immunosorbent assay (ELISA) sandwich assay

PD‐L1, PD‐L2, TIM‐3, and GAL‐9 (Cat. No. DY2045) were provided by R&D Systems. All the molecules were quantified in serum by an ELISA, compared with the corresponding standard curve, following the manufacturer's instructions. More details about kits are described in Supporting Information S1: Table S1.

### 2.8 Statistical analysis

Data were collected in Excel and analyzed in the Stata 13.1 (Stata Corp LP) and GraphPad Prism 9 (GraphPad Software). The Kolmogorov–Smirnov test was used to assess normality, and non‐parametric statistics were used.

Clinical data are presented as mean +/− standard deviation (SD) and frequency with percent. ELISA data are presented as the median and 25th–75th interquartile range (IQR) and were analyzed with the Mann–Whitney test to compare two groups or the ANOVA test adjusted by the Kruskal–Wallis method to multiple comparisons. Receiver operating characteristics analysis was generated to determine cut points, sensitivity, specificity, and the area under the curve of biomarkers.

## 3 RESULTS

### 3.1 Characteristics of enrolled post‐COVID‐ILD patients

One hundred eighteen post‐COVID‐ILD patients were divided into two groups according to lung function tests (normal and abnormal) at 1 year post‐COVID‐19. Nineteen percent (23 of 118) showed lung diffusion impairment. Both groups' demographic and clinical characteristics were compared (Table 1). Data showed that compared to the normal lung function tests group, patients with abnormal lung function were older (62 ± 11 vs. 54 ± 11, *p* = .002), predominantly women (13/23 [52%] vs. 12/23 [13%], *p* = .03) and showed lower leukocyte levels (9.6 ± 4.3 vs. 13 ± 6, *p* = .03). Other clinical data did not show statistical differences.

**Table 1:** Clinical and demographic data of post‐COVID‐ILD patients at one year of follow‐up.

	Normal lung function (*n* = 95)	Abnormal lung function (*n* = 23)	*p*‐Value
Age, years (± SD)	54 ± 11	62 ± 11	.002
Female gender (%)	12 (13)	13 (52)	.03
Type 2 diabetes mellitus (%)	34 (47)	5 (20)	.08
Systemic arterial hypertension (%)	31 (14)	5 (20)	.14
Tobacco smoking (%)	30 (33)	8 (32)	.57
Body mass index kg/m^2^ (± SD)	30 ± 6	28 ± 5	.19
Onset of symptoms, days (± SD)	11 ± 6	9 ± 4	.33
Length of hospital stay, days (± SD)	28 ± 15	34 ± 19	.07
PaO_2_/FiO_2_ (± SD)	145 ± 68	139 ± 50	.70
Fibrinogen mg/dL (± SD)	771 ± 206	735 ± 227	.51
Leukocytes c/mm^3^ (± SD)	13 ± 6	9.6 ± 4.3	.03
d‐dimer (± SD)	2.8 ± 4.9	3.8 ± 5.04	.42
Procalcitonin (± SD)	0.7 ± 1.6	0.5 ± 0.6	.56
LDH (± SD)	535 ± 259	504 ± 166	.60

*Note*: Patient groups are divided according to the behavior of lung function tests at one year post‐COVID‐19. Data are presented as mean +/− standard deviation (SD), and *n*, percentage (%).

Abbreviation: LDH, lactate dehydrogenase.

Continuous variables were tested using the Mann–Whitney *U* test, while categorical variables were compared using the Fisher exact test.

Table 2 shows the results of the pulmonary function tests and the lung alterations observed through HRCT. One year post‐COVID‐19, the group with abnormal lung tests displays lower DL_CO_ adjusted and oxygen saturation (sO2) postexercise than the normal lung tests group. HRCT showed that the patients with abnormal lung tests exhibited GGOs (*p* = .02), reticular pattern (*p* = .01), and subpleural lines (*p* = .007). Finally, the CT score was higher in the group with abnormal lung function (*p* < .001).

**Table 2:** Tomographic and lung functional evaluation of post‐COVID‐ILD patients at one year of follow‐up.

	Normal lung function (*n* = 95)	Abnormal lung function (*n* = 23)	*p*‐Value
FVC (% predicted) (± SD)	92 ± 14	87 ± 20	.34
FEV_1_ (% predicted) (± SD)	97 ± 16	95 ± 20	.61
FEV_1_/FVC (± SD)	86 ± 11	87 ± 13	.71
DL_CO_ adjusted (% predicted) (± SD)	85 ± 6	65 ± 11	<.001
6‐min walking test			
Meters	456 ± 120	397 ± 114	.07
sO2 post exercise	90 ± 2	84 ± 6	<.001
Chest HRCT			
GGO pattern (%)	10 (11)	15 (60)	.02
Reticular pattern (%)	4 (4)	11 (44)	.01
Subpleural line (%)	3 (3)	7 (28)	.007
Consolidation (%)	1 (1)	2 (8)	.21
CT score (± SD) at aCOVID time	20 ± 4	21 ± 4	.24
CT score (± SD) at follow‐up stage (one‐year post‐COVID‐19)	5 ± 5	9 ± 6	<.001

*Note*: Patient groups are divided according to the behavior of lung function tests at one year post‐COVID‐19. Data are presented as mean +/− standard deviation (SD), and *n*, percentage (%).

Abbreviations: CT, computed tomography; DLco, carbon monoxide diffusing capacity; FVC, forced vital capacity; FEV1, forced expiratory volume at the first second; GGO, ground glass opacities; HRCT, high resolution computed tomography; SD, standard deviation; sO2, oxygen saturation.

Continuous variables were tested using the Mann–Whitney *U* test, while categorical variables were compared using the Fisher exact test.

### 3.2 Increased serum PD‐L2 level 1 year post‐COVID‐19 is related to lung function and tomographic alterations

**Figure 2:**
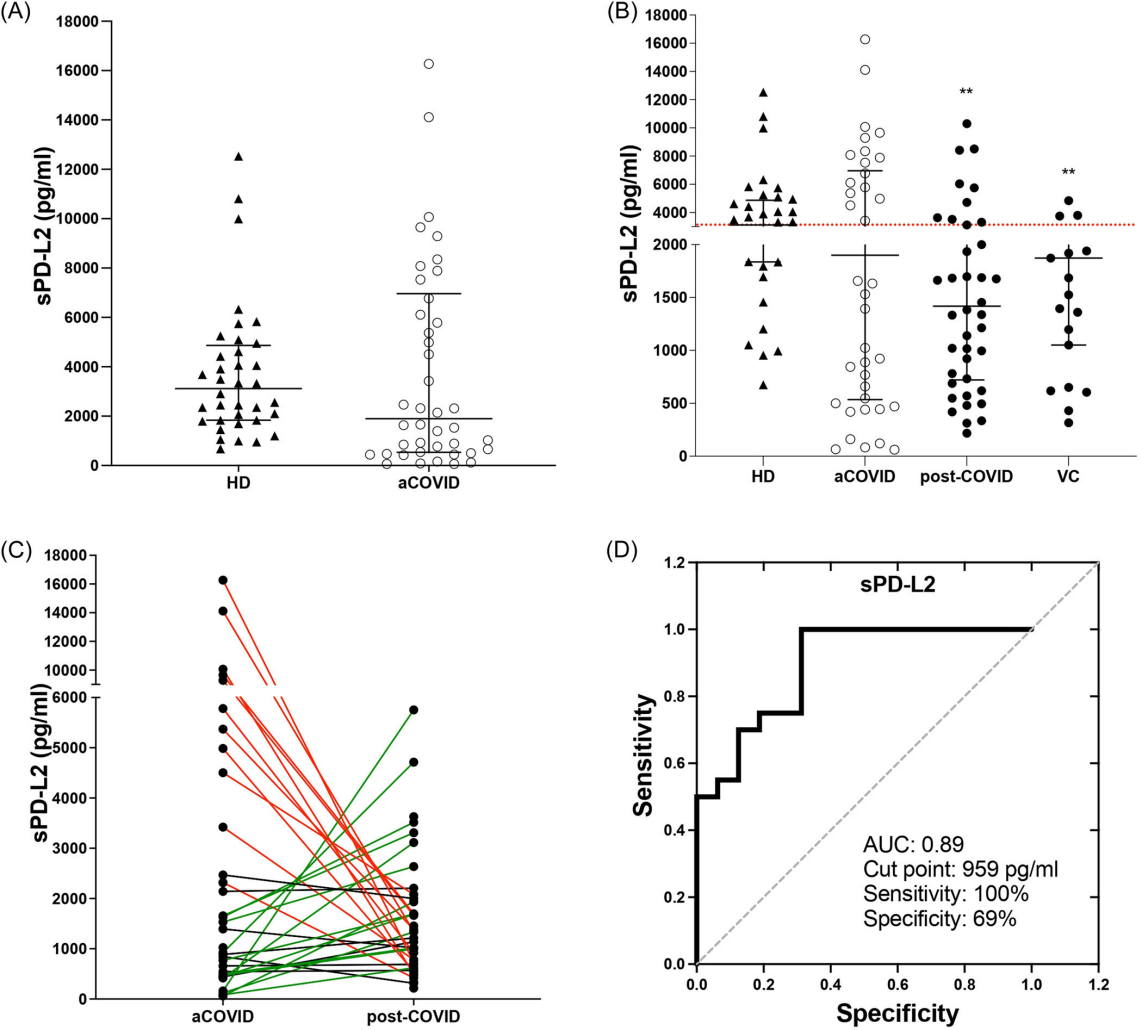
Serum PD‐L2 level at one year post‐COVID‐19 is related to persistent lung alterations. A serum sample was collected, and PD‐L2 (sPD‐L2) was measured using ELISA. (A) sPD‐L2 level in healthy donors (HD) compared to levels displayed at acute COVID‐19 (aCOVID) time from patients discharged with interstitial lung diseases (ILD) after a severe COVID‐19. (B) sPD‐L2 levels of HD, patients matched with their self aCOVID and post‐COVID‐ILD, and a verification cohort of post‐COVID‐19. (C) Behavior of sPD‐L2 level measured in 42 patients matched one‐per‐one between their aCOVID and post‐COVID‐ILD. (D) Receiver operating characteristics curves show cut points, sensitivity, specificity, and the area under the curve of PD‐L2. Graphs show individual values and median with the 25th–75th interquartile range. Statistical analysis was performed with the Mann–Whitney *U* test (to A) or ANOVA test adjusted by the Kruskal–Wallis method (to B). **p < .01, asterisks indicate a comparison to HD. Lines in (C) indicate red = those patients that decreased sPD‐L2 levels in post‐COVID‐ILD, green = those that increased, and black = those who did not show change.

The level of the PD‐L2 checkpoint was evaluated in the serum sample (hereafter called sPD‐L2) of the 42 post‐COVID‐ILD patients during the aCOVID status, and it was compared to HD. Our data showed that the sPD‐L2 level is not modified during aCOVID (aCOVID: 1899 IQR 535–6962 vs. HD: 3118 IQR 1836–4867 ng/mL) (Figure 2A). However, taking as reference the median value of serum sPD‐L2 level in HD, we observed that aCOVID could be divided into two subgroups, one that showed a value of sPD‐L2 above the median (3118 pg/mL, *n* = 21) and a second group below the median (*n* = 21).

The sPD‐L2 level was evaluated in the four groups. Data showed that post‐COVID‐ILD patients had lower levels of sPD‐L2 than HD, and this behavior was observed in both post‐COVID‐ILD and VC groups (1418 IQR 720–2755, *p* < .01; and 1872 IQR 1051–2713 ng/mL, *p* < .01; respectively) (Figure 2B). However, VC had a lower number of patients above the HD median.

We observed that the 42 patients had an ample distribution of the sPD‐L2 level compared to the VC group; to clarify how the change in the distribution of sPD‐L2 levels from aCOVID to post‐COVID‐19, arbitrarily, we established a 30% change (up or down) in post‐COVID‐ILD compared to their aCOVID levels, as a significant change.

Our data showed that a group of post‐COVID‐ILD patients did not display any change in their sPD‐L2 levels between acute and post‐COVID‐ILD stages (Figure 2C, *n* = 5, black line). Another group of patients who had a high level of sPD‐L2 at aCOVID and showed a significant decrease in post‐COVID‐ILD (Figure 2C, *n* = 19, red line); finally, another group whereas there were patients who had a low level of sPD‐L2 at aCOVID and it increased in post‐COVID‐ILD (Figure 2C, *n* = 18, green line).

We demonstrated that a 959 pg/mL cut point could be used to differentiate between post‐COVID‐ILD patients who increased sPD‐L2 levels and still displayed lung alterations versus those who decreased the sPD‐L2 level. This suggests that sPD‐L2 could be used as a follow‐up molecule for the clinical status of post‐COVID‐19 patients who show pulmonary alterations with a sensitivity of 100% and a specificity of 69% (Figure 2D).

**Figure 3:**
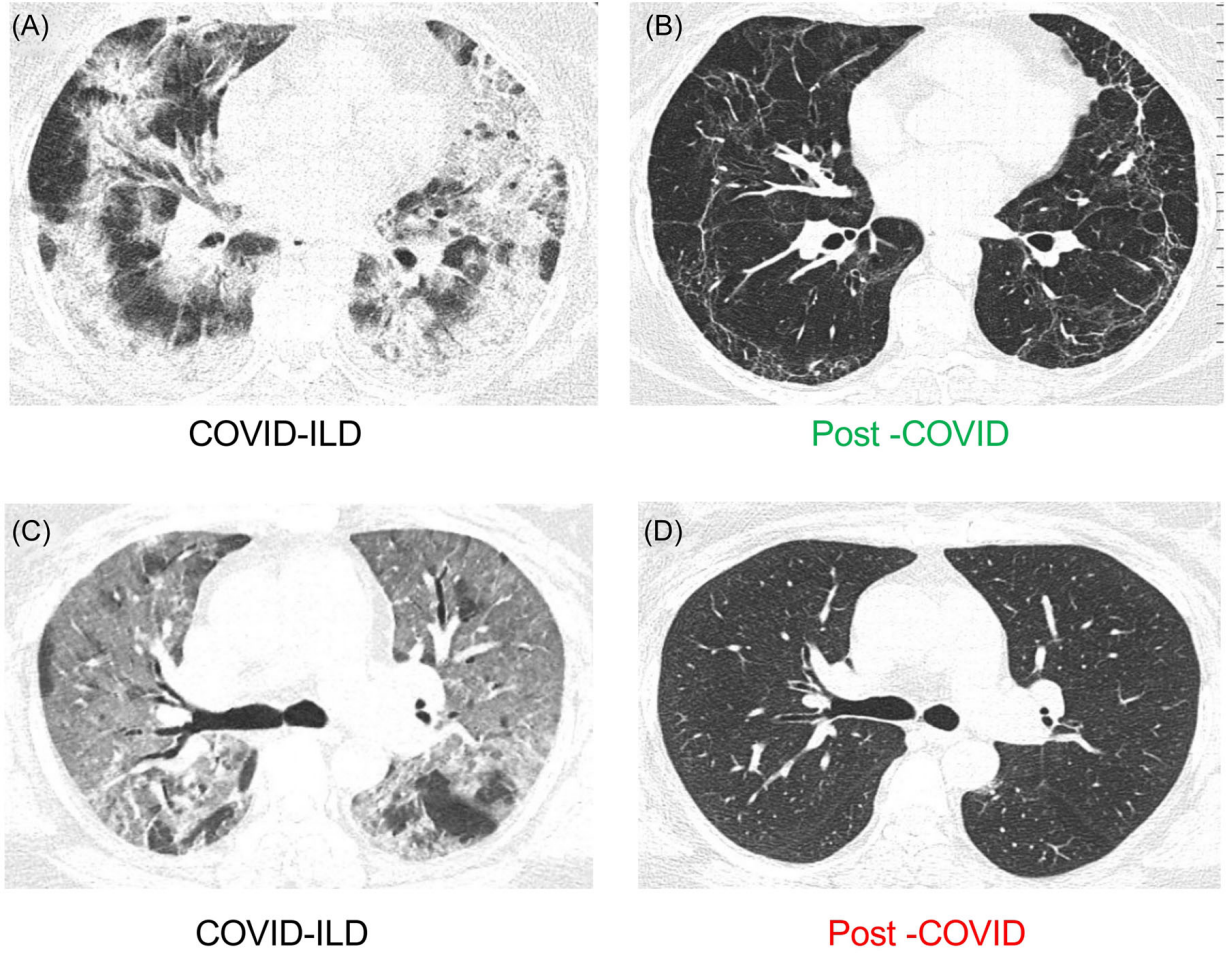
Axial images of chest high‐resolution computed tomography of two representative cases of patients discharged with interstitial lung diseases (ILD) after a severe COVID‐19, examined during acute COVID‐19 (aCOVID) and after 1‐year follow‐up (post‐COVID‐ILD). A representative patient who increased sPD‐L2 level in post‐COVID‐ILD (green color) compared to aCOVID levels. (A) HRCT acquired on the first day of hospitalization, showing an organizing pneumonia pattern. (B) HRCT of the same patient, displaying parenchymal bands, reticular pattern, and persistent ground glass opacity (GGO). A representative patient who decreased sPD‐L2 level in post‐COVID‐ILD (red color) compared to aCOVID levels. (C) HRCT acquired on the first day of hospitalization (aCOVID) exhibiting diffuse GGO as the predominant pattern. (D) HRCT of the same patient showing complete resolution of its lesion. HRCT, high‐resolution computed tomography.

Outstandingly, we confirmed that those post‐COVID‐ILD patients who displayed an increase in the level of sPD‐L2 still exhibited at 1‐year post‐COVID‐19 HRCT abnormalities, including parenchymal bands, reticular opacities, and 62% of these patients had persistent ground glass opacities (GGA). Figure 3A (from a patient during aCOVID) and Figure 3B (the same patient as A but after a 1‐year follow‐up) exemplify these findings. In sharp contrast, those post‐COVID‐ILD patients who showed a decrease in the sPD‐L2 level did not show parenchymal bands or reticular opacities (suggestive of fibrotic response), and only 33% displayed residual GGO, as illustrated in Figure 3C,D (representative imagen from the same patients during aCOVID and after 1 year, respectively).

Regarding the analysis of specific changes in lung function tests and tomographic data, our data revealed that post‐COVID‐ILD patients who increased their sPD‐L2 levels had higher CT scores than those in which PD‐L2 decreased (7.9 ± 3.4 vs. 4.2 ± 5.7, *p* = .001), and showed a lower DL_CO_ (78 ± 17 vs. 96 ± 24; *p* = .01) (Table 3). Other functional variables, such as FVC and FEV_1_, were not different between patients' subgroups.

**Table 3:** Tomographic and lung functional evaluation of post‐COVID‐ILD, classified according to the sPD‐L2 level profile.

	Up sPD‐L2 level (*n* = 18)	Down sPD‐L2 level (*n* = 19)	*p*‐Value
CT score (± SD)	7.9 ± 3.4	4.2 ± 5.7	.001
FVC (% predicted) (± SD)	82 ± 20	91 ± 16	.21
FEV_1_ (% predicted) (± SD)	89 ± 20	92 ± 15	.71
FEV_1_/FVC (± SD)	86 ± 9.31	86 ± 12	.42
DL_CO_ adjusted (% predicted) (± SD)	78 ± 17	96 ± 24	.01

*Note*: The post‐COVID‐ILD patient group is divided into those who increased or decreased 30% of their sPD‐L2 level during acute COVID‐19. Data are presented as mean +/− standard deviation (SD), and *n*, percentage (%).

Abbreviations: CT, computed tomography; DLco, carbon monoxide diffusing capacity; FVC, forced vital capacity, FEV_1_, forced expiratory volume at the first second; HRCT, high‐resolution computed tomography; ILD, interstitial lung diseases.

Continuous variables were tested using the Mann–Whitney *U* test, while categorical variables were compared using the Fisher exact test.

Clinical parameters and demographic data showed that the only difference observed between these groups of post‐COVID‐ILD patients is smoking, where 38% of patients with up sPD‐L2 levels were smokers, while in the group with down sPD‐L2 levels, only 5% (*p* = .02) (Table 4).

**Table 4:** Clinical and demographic data during acute COVID‐19 and dividing according to the sPD‐L2 level profile showed at post‐COVID‐ILD time.

	Up sPD‐L2 level (*n* = 18)	Down sPD‐L2 level (*n* = 19)	*p*‐Value
Age, years (± SD)	52 ± 15	52 ± 12	.98
Male gender (%)	12 (57)	13 (61)	1
Type 2 diabetes mellitus (%)	10 (47)	5 (23)	.1
Systemic arterial hypertension (%)	3 (14)	8 (38)	.1
Tobacco smoking (%)	8 (38)	1(5)	.02
Body mass index kg/m^2^ (± SD)	28.9 ± 5.07	30.2 ± 5.5	.41
Onset of symptoms, days (± SD)	8 ± 4	8 ± 5	.52
PaO_2_/FiO_2_ (± SD)	153 ± 62	139 ± 76	.25
Fibrinogen mg/dl (± SD)	734.4 ± 231	736.6 ± 209	.77
Leukocytes c/mm^3^ (± SD)	11.4 ± 8	11 ± 5	.9
Glucose mg/dL (± SD)	168 ± 76	146 ± 74	.3
Albumin g/dL (± SD)	3 ± 0.6	3.2 ± 0.5	.09
Alkaline phosphatase U/L (± SD)	84 ± 29	88 ± 27	.62
CT score (± SD)	20 ± 7	21 ± 4	.8

*Note*: The acute COVID‐19 patient group is divided into those who have up or down of the sPD‐L2 at post‐COVID‐ILD time. Data are presented as mean +/−  standard deviation (SD), and *n*, percentage (%).

Abbreviations: CT, computed tomography; ILD, interstitial lung diseases.

Continuous variables were tested using the Mann–Whitney *U* test, while categorical variables were compared using the Fisher exact test.

These results suggest that the increase of sPD‐L2 level in post‐COVID‐ILD patients is associated with persistent structural and functional lung alterations, which may result in a poor clinical outcome.

### 3.3 The checkpoints PD‐L1, TIM‐3, and GAL‐9 increase during acute COVID‐19, but they are downregulated at 1‐year post‐COVID‐19

**Figure 4:**
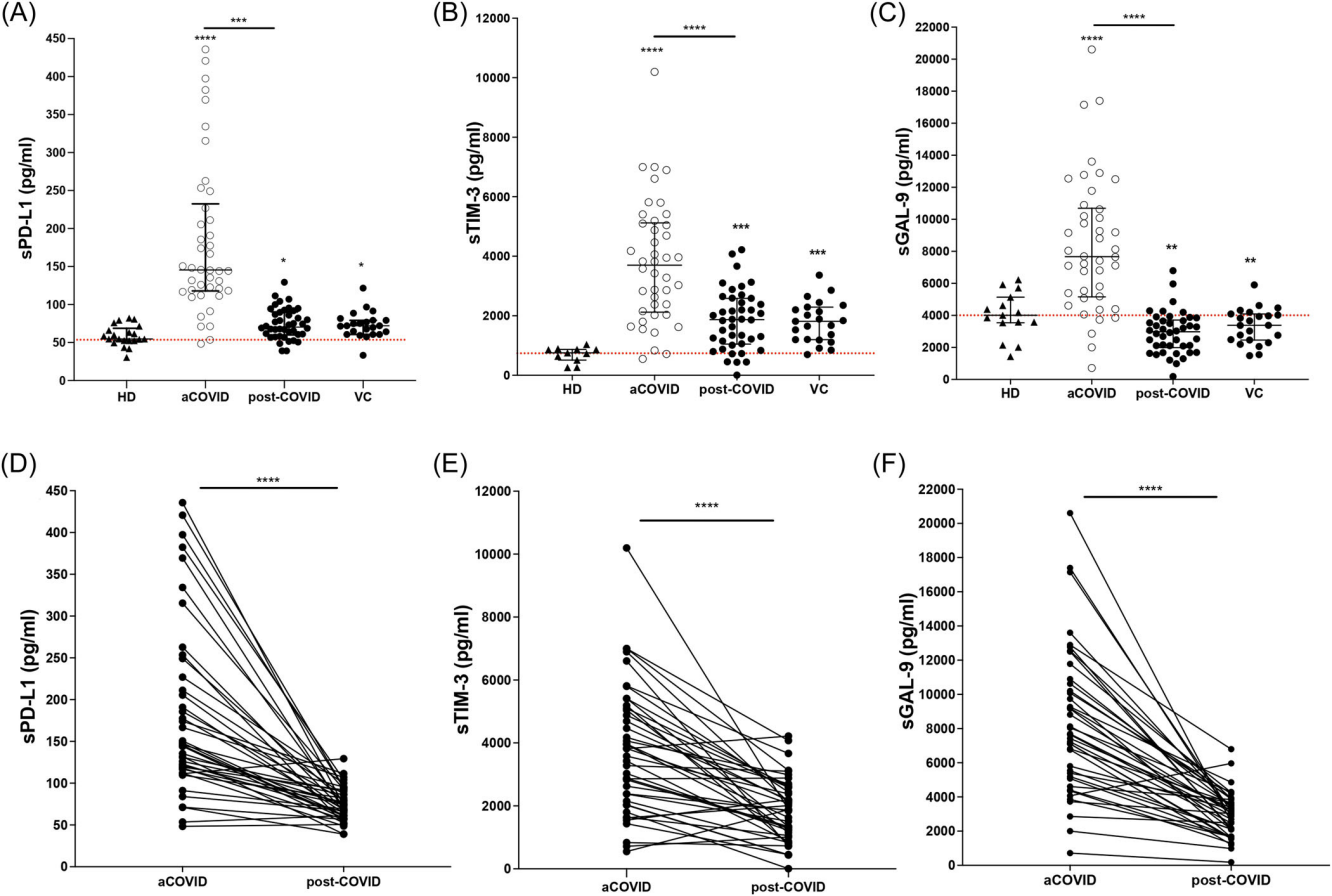
Serum levels of PD‐L1, sTIM‐3, and GAL‐9 levels increase during acute COVID‐19 and markedly decrease in post‐COVID‐ILD. Serum was collected, and levels of (A) sPD‐L1, (B) sTIM‐3, and (C) sGAL‐9 were measured using ELISA. Included groups are healthy donors (HD), acute COVID‐19 (aCOVID) and post‐COVID‐19 (one‐year) patients discharged with interstitial lung abnormalities after a severe COVID‐19 (post‐COVID‐ILD), and a verification cohort of post‐COVID‐19. The behavior of sPD‐L1 (D), sTIM‐3, and sGAL‐9 levels measured in aCOVID and after one‐year follow‐up patients matched one‐per‐one. Graphs show individual values and median with the 25th–75th interquartile range. Statistical analysis was performed with an ANOVA test adjusted by the Kruskal–Wallis method (to A–C) or the Mann–Whitney *U* test (to D–F). ***p < .001, ****p < .0001. Asterisks indicate comparison to HD; when the asterisk is on a line, it indicates the comparison between indicated groups. ELISA, enzyme‐linked immunosorbent assay.

COVID‐19 patients showed an increase in the serum PD‐L1 level (sPD‐L1) at aCOVID time compared with HD (145 IQR 60–90 vs. 55 IQR 52–69 ng/mL, respectively; *p* < .0001). At 1 year, post‐COVID‐ILD, sPD‐L1 strongly decreased, although the level was still higher than HD. This finding was observed in both post‐COVID‐ILD (70 IQR 60–90 ng/mL) and VC (72 IQR 61–79 ng/mL) groups (Figure 4A).

Similarly, the serum TIM‐3 level (sTIM‐3) increased at aCOVID compared with HD (3701 IQR 2122–5121 vs. 754 IQR 510–867 ng/mL, respectively; *p* < .0001) and at post‐COVID‐19 time, sTIM‐3 decreased, but it was still higher than HD, and this behavior was observed in both, post‐COVID‐ILD (1871 IQR 1048–2587 ng/mL) and VC (1815 IQR 1196–2292 ng/mL) groups (Figure 4B). The soluble form of GAL‐9 (sGAL‐9, ligand to TIM‐3) also increased at aCOVID compared to HD (7669 IQR 5161–10690 vs. 4,010 IQR 3546–5138 ng/mL, respectively; *p* < .0001), but after 1 year, the GAL‐9 level decreased and the level was even lower than HD, in both post‐COVID‐ILD (2979 IQR 1988–3712 ng/mL) and VC (3380 IQR 2462–4093 ng/mL) groups (Figure 4C).

As illustrated in Figure 4D–F, the longitudinal follow‐up of checkpoint levels per patient showed a strong and similar downregulation of sPD‐L1, sTIM‐3, and sGAL‐9 1 year post‐COVID‐19.

### 3.4 Serum levels of PD‐L1 and TIM‐3 show high sensitivity and specificity to differentiate acute COVID‐19

To identify a complementary tool for the COVID‐19 diagnosis, we evaluated whether the levels of sPD‐L1, sTIM‐3, and sGAL‐9 can be used to improve the battery of current biomarkers options.

**Figure 5:**
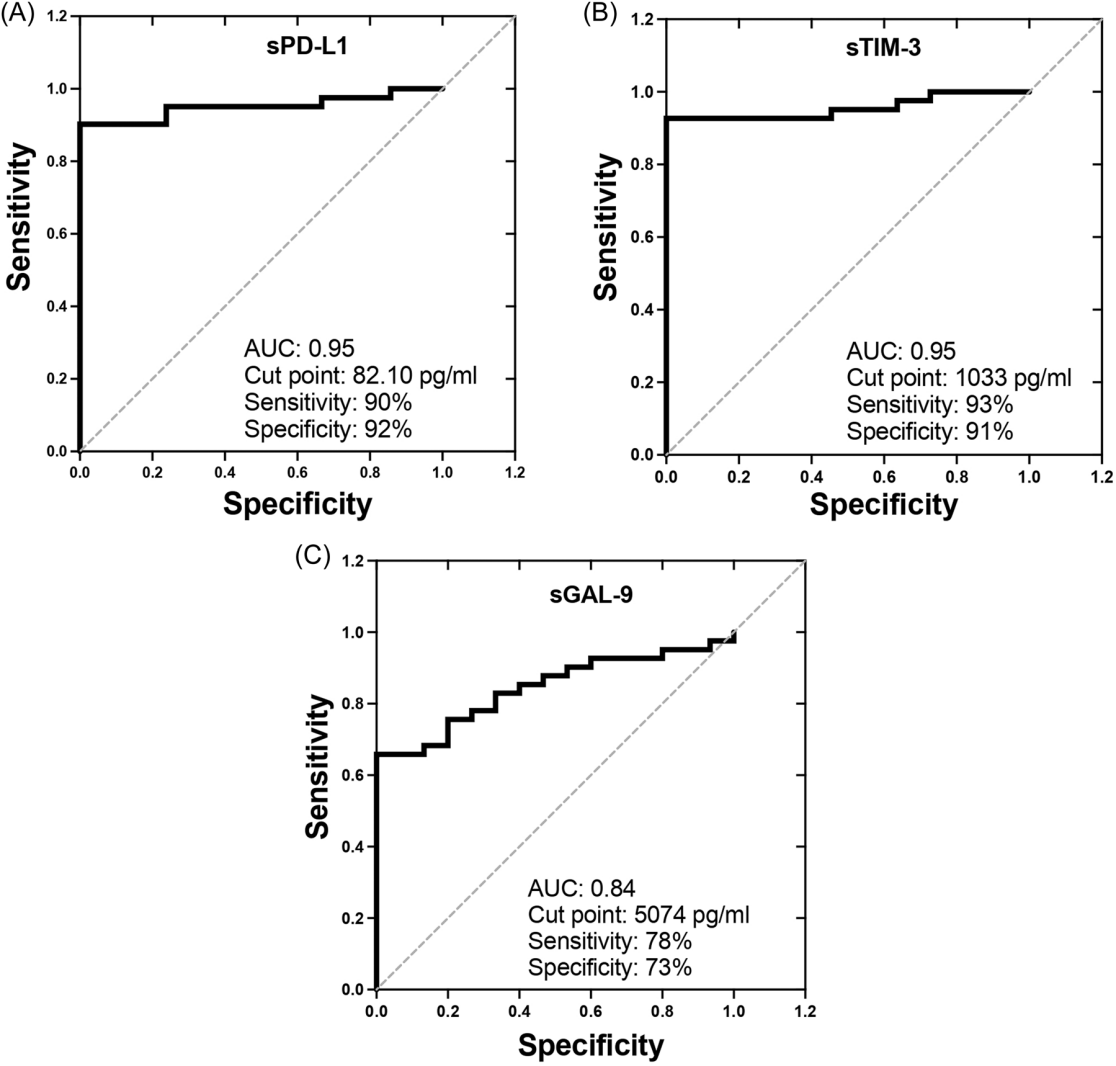
Serum levels of sPD‐L1 and sTIM‐3 can be complementary tools for the COVID‐19 diagnosis. Receiver operating characteristics curves show cut points, sensitivity, specificity, and the area under the curve of biomarkers (A) sPD‐L1, (B) sTIM‐3, and (C) sGAL‐9 to differentiate into COVID‐19 patients versus healthy donors.

Our data showed that 82.1 pg/mL of sPD‐L1 can be used as a cut point to differentiate between HD and aCOVID patients with a sensitivity of 90% and specificity of 92% (Figure 5A). Likewise, 1033 pg/mL of sTIM‐3 showed a sensitivity of 93% and specificity of 91% (Figure 5B), whereas the sGAL‐9 level (cut point of 5074 pg/mL) showed only a sensitivity of 78% and specificity of 73% (Figure 5C).

### 3.5 Post‐COVID‐ILD patients with a up‐sPD‐L2 level profile also have altered the CD4/CD8 ratio

T‐cells are one of the leading players in the adaptive immune response. To confirm if those who express the transmembrane form of PD‐L1 and PD‐L2 are altered in up‐sPD‐L2 post‐COVID‐ILD patients, 10 patients up‐ and 10 down‐sPD‐L2 levels from our post‐COVID‐ILD group were aleatory selected, and a phenotypical characterization by flow cytometry was performed.

**Figure 6:**
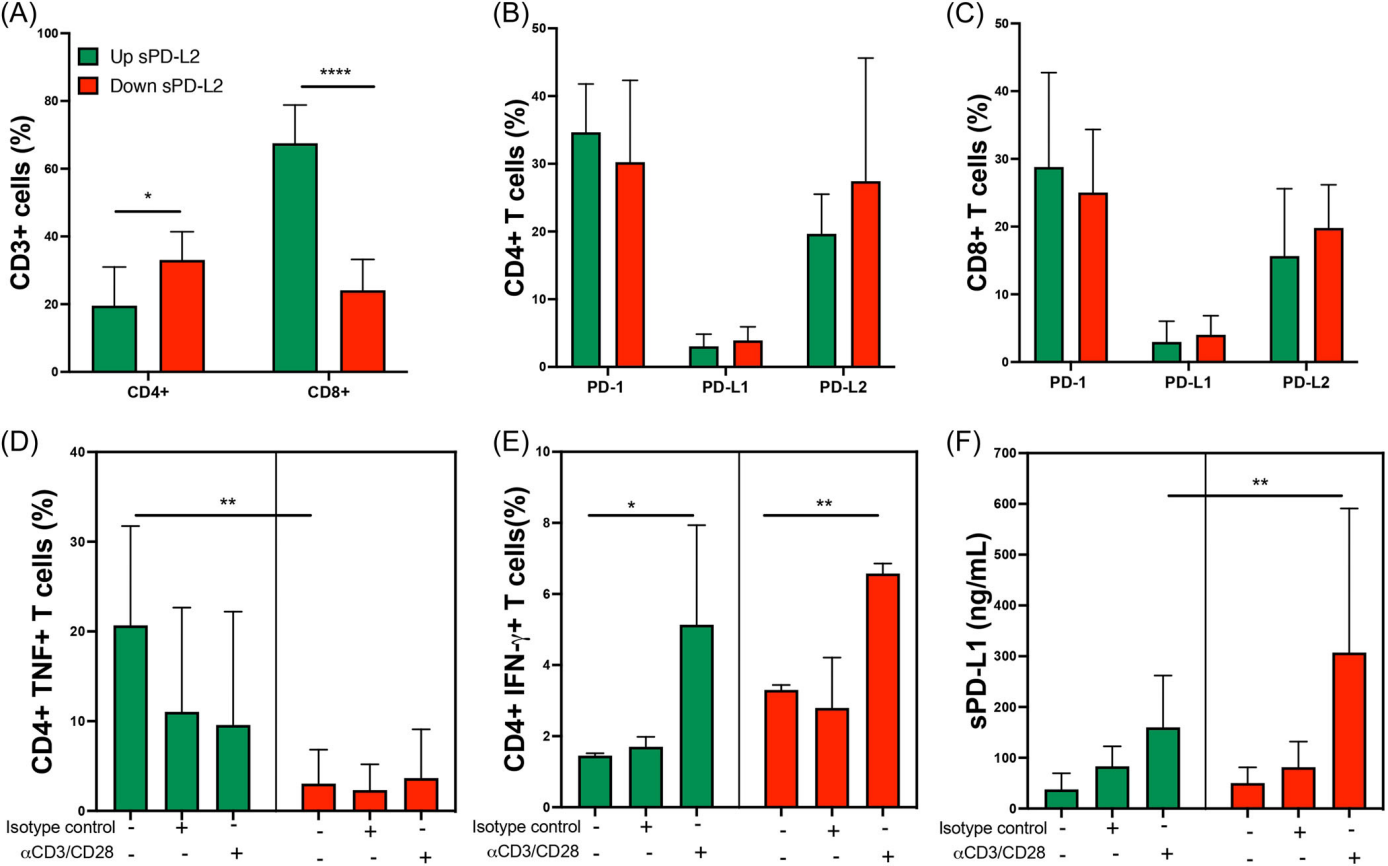
Post‐COVID‐ILD patients with an up‐sPD‐L2 profile have altered the CD4/CD8 ratio. Mononuclear cells from patients post‐COVID‐ILD that increased (up) or decreased (down) their serum levels of sPD‐L2 were stimulated with anti‐CD3 and anti‐CD28 (αCD3/CD28), as negative controls were used cells without stimulus and with isotype controls, cells were prepared to flow cytometry and supernatant was used to evaluate soluble molecules. (A) The frequency of CD4+ and CD8+ was evaluated into the CD3+ gate (T‐cells). The frequency of CD4+ T‐cells (B) and CD8+ T‐cells (C) positive to PD‐1, PD‐L1, and PD‐L2 is shown. The frequency of CD4+TNF+ T‐cells (D) and CD4+IFN‐γ+ (E) T‐cells was obtained. The soluble PD‐L1(sPD‐L1) was measured in the culture supernatant by ELISA (F). Graphs show the median with the 25th–75th interquartile range. Statistical analysis was performed with an ANOVA test adjusted by the Kruskal–Wallis. *p < .05, **p < .01, ****p < .0001, and asterisks indicate comparison between indicated groups. ELISA, enzyme‐linked immunosorbent assay.

Data showed that up‐sPD‐L2 patients have decreased the frequency of CD4+ and increased CD8+ T‐cells compared to those with down‐sPD‐L2 levels (Figure 6A). Thus, up‐sPD‐L2 has a CD4/CD8 ratio of 0.3, whereas the down‐sPD‐L2 has a ratio of 1.7, a similar value than reported to healthy (the ratio should be greater than 1). Surprisingly, the frequency of CD4+ T‐cells positive to PD‐1, PD‐L1, and PD‐L2 was not different between groups (Figure 6B), and the same result was observed for CD8+ T‐cells (Figure 6C).

Based on the CCR7 and CD45RA, we evaluated if the subsets naïve, effector or central memory (EM and CM, respectively) and effector memory cells re‐expressing CD45RA (TEMRA) are modified in T‐cells (Supporting Information S1: Figure S2A); however, we did not identify differences in naïve, EM, CM, and TEMRA subsets both into the gate of CD4+ and CD8+ T‐cells (Supporting Information S1: Figure S2B,C).

Following, to clarify if the function of CD4+ (cytokines secretion) or CD8+ (cytotoxic molecules) T‐cells are modified, mononuclear cells from both post‐COVID‐ILD patient groups were cultured and stimulated with anti‐CD3 and ‐CD28. We observed that under basal conditions, the up‐sPD‐L2 group has a higher frequency of CD4+TNF+ T‐cells compared to the down‐sPD‐L2 group (Figure 6D). The frequency of CD4+IFN‐γ+ T‐cells increases after a polyclonal stimulus, and this comportment was observed in both groups (Figure 6E). The frequency of CD8+ T‐cells positive to the cytotoxic molecules Granzyme B and Perforin did not show any differences at basal (Supporting Information S1: Figure S3) or under stimulated conditions (data are not shown).

Finally, soluble levels of sPD‐L1 and sPD‐L2 in the culture supernatant were evaluated. Our data showed that cells from the down‐sPD‐L2 group are higher producers of sPD‐L1 than the up‐sPD‐L2 group when receiving a polyclonal stimulus, although, at baseline, both groups produce similar sPD‐L1 levels (Figure 6E). In consonance with the phenotypical characterization of T‐cells, sPD‐L2 levels were not different between groups, and it is maintained even after a polyclonal stimulus (Supporting Information S1: Figure S4).

## 4 DISCUSSION

Our results demonstrated that most patients discharged with residual lung abnormalities compatible with interstitial lung disease after an aCOVID normalized their lung function at 1 year post‐COVID‐19. However, 12% of post‐COVID‐ILD patients still showed functional and HRCT alterations. In this context, we found that those with increased (at least 30%) sPD‐L2 levels and altered CD4/CD8 ratio at post‐COVID‐19 time exhibited persistence of chest tomographic and functional lung alterations. In contrast, those with decreased sPD‐L2 levels displayed a complete lung recovery. Other molecules such as sPD‐L1, sTIM‐3, and sGAL‐9 significantly increased during aCOVID but decreased in all patients after 1‐year follow‐up.

The post‐COVID‐19 syndrome comprises a wide range of physical and mental health symptoms that persist after recovery from an acute SARS‐CoV‐2 infection.^2^, ^17^ In acute critical COVID‐19, hyperinflammation and endothelial dysfunction are present; corticosteroid therapy has been the first‐line treatment since the findings of the RECOVERY trial.^18^ The post‐mortem histopathological analyses have suggested persisting fibrotic abnormalities; however, as discussed by Ntatsoulis et al., large‐scale, long‐term follow‐up studies are necessary to clarify the link between COVID‐19 and interstitial lung alterations because, on the one hand, SARS‐CoV‐2 infection induces pro‐fibrotic factors such as the transforming growth factor‐β1 (TGFβ1). On the other hand, ILDs have increased odds of ARDS development and severe COVID‐19.^19^

Limited studies help establish biomolecules for better monitoring of post‐COVID‐19 patients, mainly those sequelae where lung function is compromised; some studies showed that 1–3 months after discharge, patients report residual impairment of primarily DL_CO_ and, secondly, FVC.^3^, ^20^, ^21^ However, in a long‐term follow‐up of patients with MERS, slightly over one‐third of the patients had impaired DL_CO_ at a 15‐year follow‐up.^22^

We found that 19% of COVID‐19 patients still have evidence of defect DL_CO_ and sO2 postexercise 1 year after discharge. Low DL_CO_ could be caused by interstitial changes or pulmonary vascular abnormalities following COVID‐19 infections. Accordingly, these patients persist with several tomographic lung abnormalities and have a CT score higher than those with normal lung functional tests.

The immune checkpoint mediated by PD‐1 and its ligands has been widely studied in the cancer field, but its role in no‐tumor cells is poorly explored. The development of inhibitors to the PD‐1 pathway is efficient in restoring the T cell function; however, several of these inhibitors focus on the PD‐L1 ligand, and recently, it was proposed that PD‐L1 may mediate pulmonary fibrosis.^23^, ^24^

Despite PD‐L1 and PD‐L2 being ligands to the same receptor, the PD‐L1 and PD‐L2 expression are differentially regulated. PD‐L1 is upregulated by toll‐like receptor‐4 signal transducer and activator of transcription 1 (STAT1) dependent signaling. In contrast, the PD‐L2 expression depends on the interleukin 4 alpha receptor (IL‐4Rα) and STAT6,^25^, ^26^ suggesting that the microenvironment is relevant to determining which ligand increases.

In this study, we analyzed the levels of immune checkpoints PD‐L1, PD‐L2, TIM‐3, and Gal‐9 at post‐COVID‐19 and acute COVID‐19 times. Unlike the other checkpoints, PD‐L2 was not increased during acute COVID‐19, but a small group of patients showing very low PD‐L2 levels presented an increase at 1 year and persisted with structural and functional pulmonary alterations.

The reasons why PD‐L2 behaves so differently to PD‐L1 are unclear. Due to the PD‐L2 binds to PD‐1 with stronger affinity than PD‐L1,^27^ probably during acute COVID‐19, the immune suppression is mediated mainly by PD‐L1 in COVID‐ILD patients, and it does not happen during post‐COVID‐19. Considering that PD‐L1 and PD‐L2 are differently regulated, another possibility is that the microenvironment generated at acute COVID‐19 versus post‐COVID‐19 modulates the presence of only one ligand. As discussed below, the absence or the increase of PD‐L2 mediates diverse mechanisms.

Our data indicate that PD‐L2 level allows differentiating the COVID‐ILD patients mainly into two groups post‐COVID‐19: those patients who increased the PD‐L2 level (concerning their acute COVID‐19 level) and still display lung alterations and those who decreased, suggesting that PD‐L2 level can be used to determine if COVID‐ILD patients will maintain for long time lung alterations. However, further studies are required to clarify if the negative effect on the respiratory system is because during acute COVID‐19, the COVID‐ILD patients had a very low level of PD‐L2 or because it was increased post‐COVID‐19.

In this regard, it has been reported that PD‐L2 deficiency prevents the induction of respiratory tolerance, an immune process necessary to prevent the inappropriate activation of CD4+ T cells.^28^ Recently, it was demonstrated that PD‐L2 regulates the differentiation of regulatory T cells (Treg); thus, the absence of PD‐L2 destabilizes the nuclear factor Foxp3, which is necessary to maintain Treg, suggesting that the expression of PD‐L2 is necessary for the development of the immunoregulation and tolerance.^29^ In this context, we can speculate that the decrease or lack of response of PD‐L2 during the acute phase of COVID‐19 may contribute to the immune dysfunction that characterizes this disease, which is observed by the altered presence of CD4+ T‐cells in post‐COVID‐ILD patients with a profile of up‐sPDL‐2 levels and consequently, may disturb the systemic or local microenvironment of cytokines. Of note is that these patients showed a significant increase of CD8+ T‐cells, but these cells did not have alterations in the presence of cytotoxic molecules.

CD4+ T‐cells are one of the leading players in driving the adaptive immune response, primarily secreting cytokines to regulate or activate other cell subsets. Different studies have demonstrated that specific CD4+ T‐cell subsets are necessary to alleviate some interstitial lung damage or maintain IFN‐γ production, avoiding senescence‐associated pulmonary fibrosis.^30^, ^31^

The up‐sPD‐L2 profile observed in those post‐COVID‐ILD patients with lung structural and functional alterations could affect other cell subsets; reports indicated that high expression of PD‐L2 is related to the presence of a macrophage subpopulation with anti‐inflammatory and profibrotic functions called M2, which leads to the inhibition of T‐cells, and in tumor models has been described that high expression of PD‐L2 is associated with a poor prognosis.^32^, ^33^ Several studies suggest that M2 macrophages play a central role in the pathogenesis of fibrosis; for instance, M2 secretes connective tissue growth factors to mediate the proliferation and migration of fibroblasts.^34^ Similarly, the depletion of the gen Mbd2 attenuates the TGF‐β1 production and reduces M2 macrophage accumulation in lung fibrosis.^35^ Interestingly, one of the main research lines in developing new pulmonary fibrosis treatment schemes is avoiding the M2 macrophage differentiation.^36^, ^37^ Thus, this study opens a new question about the relation between PD‐L2/macrophages/lung alterations in post‐COVID‐19 patients.

The potential link between PD‐L2 and Gal‐9 concerning the polarization of M2 macrophages is an intriguing and relevant topic that warrants further investigation, given the relevance of M2 macrophage polarization in respiratory diseases, including pulmonary fibrosis. We conducted a correlation analysis between PD‐L2 and Gal‐9, and logistic regressions were performed, but we could not identify significant correlations between variables and PD‐L2 levels. Our study is not free of limitations, and the small sample size may influence not observing correlations. Moreover, this COVID‐19 cohort includes only severe patients, limiting the results' interpretation to mild or moderate disease.

Our study suggests that several immune checkpoints like PD‐L1, TIM‐3, and GAL‐9 strongly increase in acute COVID‐19 but decrease and virtually normalize during follow‐up, suggesting that they could be good tools to improve the current options battery for COVID‐19 diagnosis. In contrast, PD‐L2 is not increased at acute COVID‐19 time, but its pattern of systemic level could be a good biomarker to follow in post‐COVID‐19 to evaluate the persistence of lung damage in COVID‐ILD patients.

## 5 CONCLUSION

Circulating levels of sPD‐L2 together with an altered CD4/CD8 ratio could be used as indicative of the persistence of lung lesions post‐COVID‐19 in COVID‐ILD patients, although sPD‐L1 could be helpful to improve the diagnosis during acute COVID‐19.

## AUTHOR CONTRIBUTIONS

**Ivette Buendia‐Roldan**: Conceptualization; methodology; resources; writing—original draft. **Karen Martínez‐Espinosa**, **Maria‐Jose Aguirre**, **Hiram Aguilar‐Duran**, **Alexia Palma‐Lopez**, **Yadira Palacios**, **Andy Ruiz**, and **Ranferi Ocaña‐Guzmán**: Methodology. **Lucero A Ramón‐Luing, Gloria Pérez‐Rubio**, and **Ramcés Falfán‐Valencia**: Methodology; validation. **Moisés Selman**: Conceptualization; methodology; writing—review & editing. **Leslie Chavez‐Galan**: Conceptualization; formal analysis; methodology; resources; supervision; writing—review & editing.

## CONFLICT OF INTEREST STATEMENT

The authors declare that the research was conducted in the absence of any commercial or financial relationships that could be construed as a potential conflict of interest.

## ETHICS STATEMENT

The studies involved human participants, and it was reviewed and approved by the ethical committee of the Instituto Nacional de Enfermedades Respiratorias Ismael Cosio Villegas (INER, Protocol numbers C53‐20 and B04‐22). The patients provided their written informed consent to participate in this study.

## Supporting information

Supporting information.

## Data Availability

The original contributions presented in the study are included in the article and supplementary material. Further inquiries can be directed to the corresponding author.
