# Supplementary material for: Persistence of lung structural and functional alterations at one year post‐COVID‐19 is associated with increased serum PD‐L2 levels and altered CD4/CD8 ratio
Source: Immun Inflamm Dis. 2024 Jul 19;12(7):e1305. doi: 10.1002/iid3.1305 (PMC11259001; doi:10.1002/iid3.1305)
Supplement: Supplementary file 1 — Supporting information. [file IID3-12-e1305-s001.docx]

**Persistence of lung structural and functional alterations at one year post-COVID-19 is associated with increased serum PD-L2 levels and altered CD4/CD8 ratio**

Ivette Buendia-Roldan^a^, Karen Martínez-Espinosa^a^, Maria-Jose Aguirre^a^, Hiram Aguilar-Duran^a^, Alexia Palma-Lopez^a^, Yadira Palacios^a^, Andy Ruiz^a^, Lucero A. Ramón-Luing^a^, Ranferi Ocaña-Guzmán^a^, Gloria Perez-Rubio^a^, Ramcés Falfán-Valencia^a^, Moisés Selman^a^, Leslie Chavez-Galan^a*^.

Supplementary material

**Table S1**. Antibodies used in the present study.

| Anti- | Fluorochrome | Use to | Company | Catalog/clone |
| --- | --- | --- | --- | --- |
| CD3 | NA | Culture | BIOLEGEND | 317315/OKT3 |
| CD28 | NA | Culture | BIOLEGEND | 302923/CD28.2 |
| IgG2 | NA | Isotype Control | BD Pharmingen | 554645 |
| CD3 | PE | Flow cytometry | BIOLEGEND | 981004/SK7 |
| CD28 | FITC | Flow cytometry | BIOLEGEND | 122008/E18 |
| CD8 | FITC | Flow cytometry | BIOLEGEND | 301050/RPA-T8 |
| CD4 | Brilliant Violet 510 | Flow cytometry | BIOLEGEND | 300546/RPA-T4 |
| CD45RA | APC | Flow cytometry | BIOLEGEND | 304112/HI100 |
| PD-1 | PE-Cyanine7 | Flow cytometry | BIOLEGEND | 329918/EH12.2H7 |
| PD-L2 | APC/Cyanine7 | Flow cytometry | BIOLEGEND | 345516/MIH18 |
| CCR7 | PerCP | Flow cytometry | BIOLEGEND | 353242/G043H7 |
| Granzyme B | PE-Cyanine5 | Flow cytometry | BIOLEGEND | 372226/QA16A02 |
| Perforin | APC | Flow cytometry | BIOLEGEND | 353312/B-D48 |
| CD45RA | APC | Flow cytometry | BIOLEGEND | 304112/HI100 |
| IFN- 𝛾 | FITC | Flow cytometry | BIOLEGEND | 502506/4S.B3 |
| TNF- 𝛼 | APC | Flow cytometry | BIOLEGEND | 502902/MAB11 |
| PD-L1 | NA | ELISA | R&D | DY156 |
| PD-L2 | NA | ELISA | R&D | DY1224 |
| TIM-3 | NA | ELISA | R&D | DY2365 |
| GAL-9 | NA | ELISA | R&D | DY2045 |

**Figures**


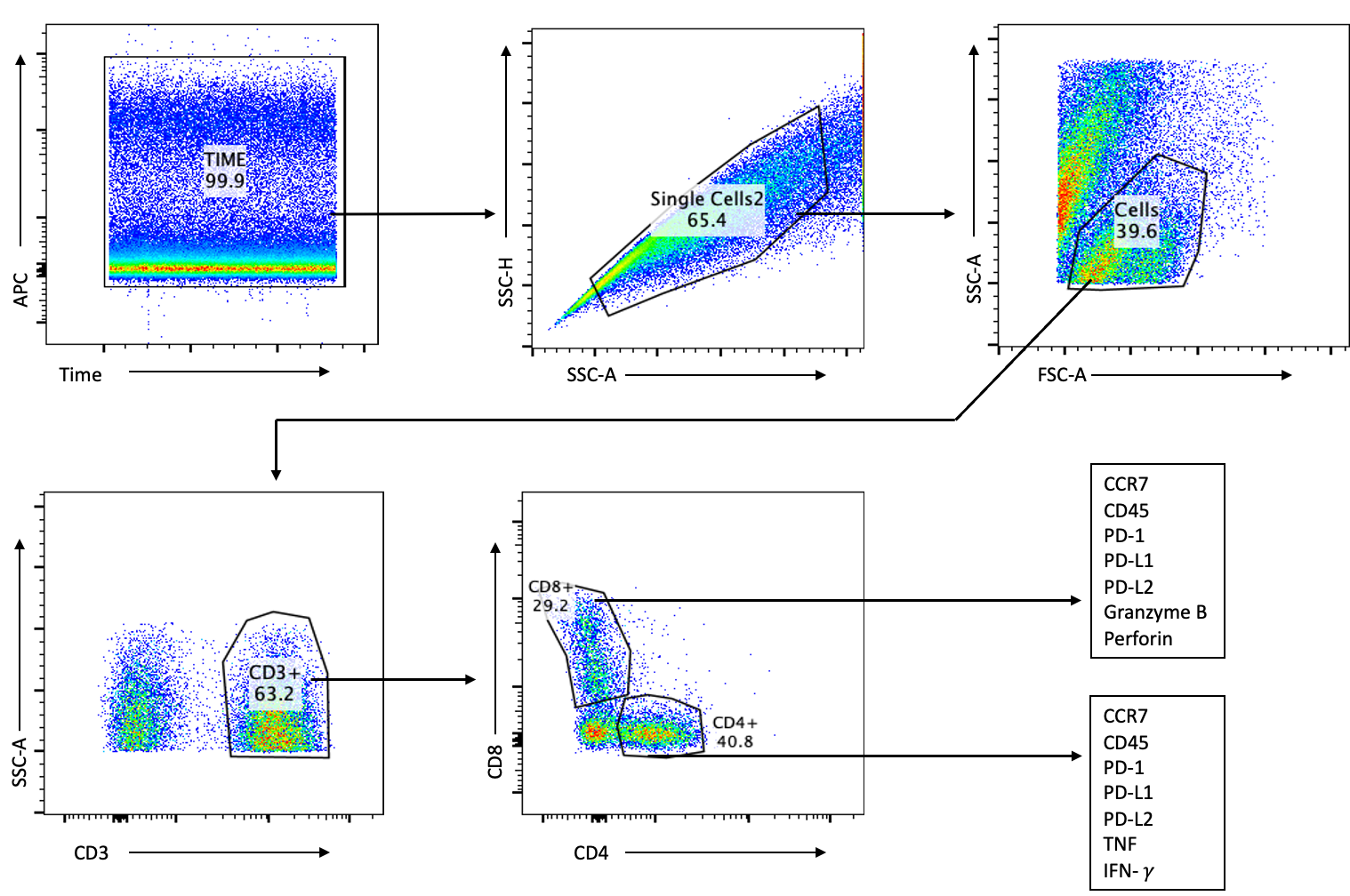


**Figure S1. Flow Cytometry Analysis: Representative Dot Plots Showing Gating Analysis.** The analysis served as a quality control measure to identify and eliminate anomalies caused by flow rate variations using the APC channel and Time parameters (upper left). Singlet cells were chosen based on single events identified by side scatter (SSC-H vs. SSC-A) dot plot (upper center). Cells were selected based on normal size using forward scatter and side scatter (upper right). Total lymphocytes were identified by CD3 expression (bottom left), and lymphocyte subtypes, such as helper and cytotoxic cells, were distinguished using CD4 and CD8 markers, respectively (bottom center). In each subpopulation, all the molecules of interest were evaluated in each type of lymphocyte (black rectangles).


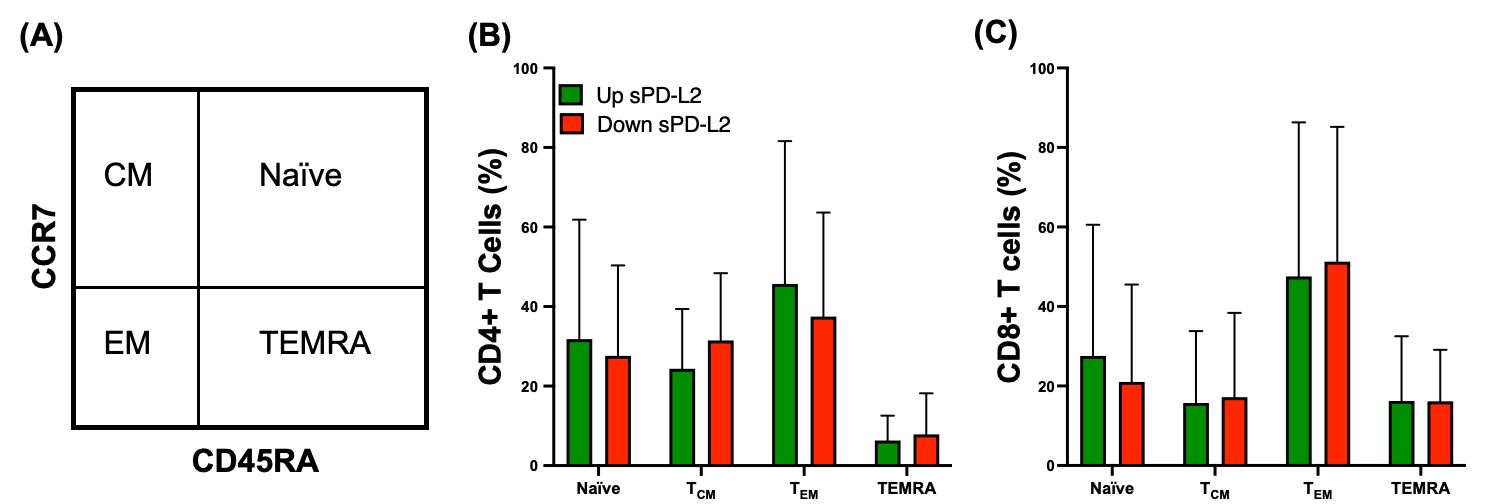


Figure S2. Distribution of CD4+ and CD8+ T cell subsets. Mononuclear cells from patients post-COVID (one year) that increased (up) or decreased (down) their serum levels of sPD-L2 were used to evaluate the frequency of the subsets naïve, effector, or central memory (EM and CM, respectively) and effector memory cells re-expressing CD45RA (TEMRA) by flow cytometry, based on the expression of CCR7 and CD45RA (A). Frequency of Naïve, CM, EM, and TEMRA subsets into the gate of CD4+ T cells (B). Frequency of Naïve, CM, EM, and TEMRA subsets into the gate of CD8+ T cells (C). Graphs show the median with the 25th-75th interquartile range (IQR).


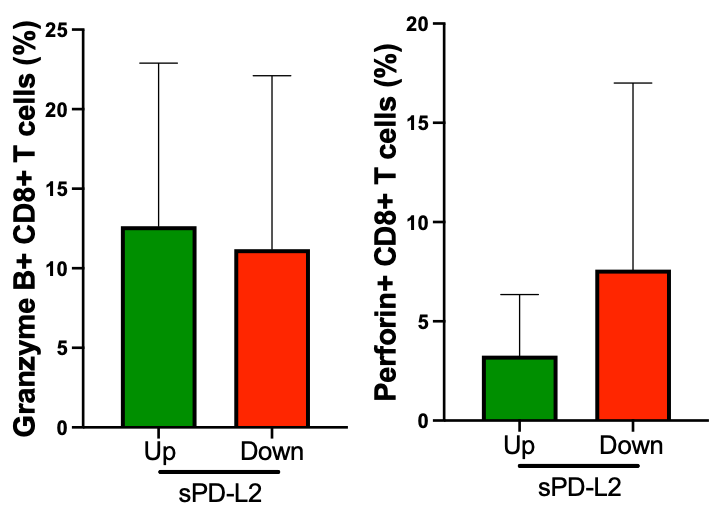


Figure S3. Expression of Granzyme B and Perforin in CD8+ T cell. Mononuclear cells from patients post-COVID (one year) that increased (up) or decreased (down) their serum levels of sPD-L2 were used to evaluate the frequency of CD8+ T cells positive to Granzyme B and Perforin. Graphs show the median with the 25th-75th interquartile range (IQR).


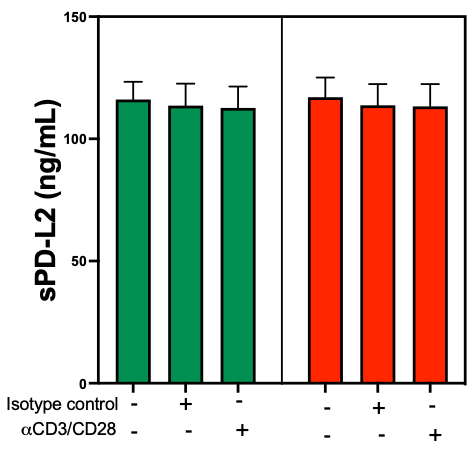


Figure S4. Soluble levels of PD-L2 in the culture supernatant subsets. Mononuclear cells from patients post-COVID (one year) that increased (up, green) or decreased (down, red) their serum levels of sPD-L2 were stimulated with anti-CD3 and anti-CD28 (αCD3/CD28), as negative controls were used cells without stimulus and with isotype controls. The supernatant was recovered and used to evaluate sPD-L2 by ELISA. Graphs show the median with the 25th-75th interquartile range (IQR).
